# Addressing a silent and neglected scourge in sexual and reproductive health in Sub-Saharan Africa by development of training competencies to improve prevention, diagnosis, and treatment of female genital schistosomiasis (FGS) for health workers

**DOI:** 10.1186/s12978-021-01252-2

**Published:** 2022-01-24

**Authors:** Julie Jacobson, Anastasia Pantelias, Megan Williamson, Eyrun Floerecke Kjetland, Alison Krentel, Margaret Gyapong, Pamela Sabina Mbabazi, Amadou Garba Djirmay

**Affiliations:** 1 Bridges to Development, Washington, USA; 2 University of Geneva, Geneva, Switzerland; 3 Discipline of Public Health Medicine, Nelson R Mandela School of Medicine, College of Health Sciences, University of KwaZulu-Natal, Durban, 4041 South Africa; 4 School of Epidemiology and Public Health, Faculty of Medicine, University of Ottawa and Bruyère Research Institute, Ottawa, Canada; 5 Institute of Health Research, University of Health and Allied Sciences, Ho, Ghana; 6 Department of Control of Neglected Tropical Diseases, World Health Organization, Geneva, Switzerland; 7 Norwegian Centre for Imported and Tropical Diseases, Department of Infectious Diseases Ullevaal, Oslo University Hospital, 0450 Oslo, Norway

**Keywords:** Female genital schistosomiasis (FGS), Schistosomiasis, Neglected tropical diseases (NTD), Reproductive health, Health equity, HIV, Cervical cancer, Colposcopy, Sub-Saharan Africa

## Abstract

**Background:**

Schistosomiasis is an acute and chronic disease caused by parasitic worms, that can take two main forms: intestinal or urogenital. If left untreated, the urogenital form can lead to female genital schistosomiasis (FGS) in women and girls; frequently resulting in severe reproductive health complications which are often misdiagnosed as sexually-transmitted infections (STIs) or can be confused with cervical cancer. Despite its impact on women’s reproductive health, FGS is typically overlooked in medical training and remains poorly recognized with low awareness both in affected communities and in health professionals. FGS has been described as the one of the most neglected sexual and reproductive health issues in sub-Saharan Africa (Swai in BMC Infect Dis 6:134, 2006; Kukula in PLoS Negl Trop Dis 13:e0007207; Joint United Nations Programme on HIV/AIDS (UNAIDS) 2019). Increased knowledge and awareness of FGS is required to end this neglect, improve women’s reproductive health, and decrease the burden of this preventable and treatable neglected tropical disease.

**Methods:**

We conducted interactive virtual workshops, in collaboration with the World Health Organization (WHO), engaging 64 participants with medical and public health backgrounds from around the world to establish standardized skills (or competencies) for prevention, diagnosis, and treatment of FGS at all levels of the health system. The competencies were drafted in small groups, peer-reviewed, and finalized by participants.

**Results:**

This participatory process led to identification of 27 skills needed for FGS prevention, diagnosis, and management for two categories of health workers; those working in a clinical setting, and those working in a community setting. Among them, ten relate to the diagnosis of FGS including three that involve a pelvic exam and seven that do not. Six constitute the appropriate behaviors required to treat FGS in a clinical setting. Eleven address the community setting, with six relating to the identification of women at risk and five relating to prevention.

**Conclusion:**

Defining the skills necessary for FGS management is a critical step to prepare for proper diagnosis and treatment of women and girls in sub-Saharan Africa by trained health professionals. The suggested competencies can now serve as the foundation to create educative tools and curricula to better train health care workers on the prevention, diagnosis, and management of FGS.

**Supplementary Information:**

The online version contains supplementary material available at 10.1186/s12978-021-01252-2.

## Plain Language Summary

Schistosomiasis belongs to the group of neglected tropical diseases (NTDs) found in tropical and subtropical countries, disproportionately affecting poor populations with limited access to safe water and adequate sanitation. Female genital schistosomiasis (FGS) is a chronic disease caused by parasitic worms, schistososomes, transmitted by contact with infested fresh water. When left untreated, FGS can lead to severe reproductive health complications, such as sub-or infertility and ectopic pregnancy. FGS is a leading neglected issue in sexual and reproductive health in sub-Saharan Africa; however, health professionals are not familiar with it, leading to misdiagnosis and improper treatment. A critical challenge in addressing the burden of FGS is the knowledge gap about the disease in communities and health professionals in endemic areas and the world at large. As part of an FGS Accelerated Scale Together (FAST) package, and in an attempt to address the knowledge gap in health professionals, we organized an interactive workshop bringing together a group of 64 participants working in sexual and reproductive health generally, and/or FGS specifically. Together, we developed a comprehensive set of required skills for health workers training on FGS. The workshop participants drafted 27 skills (or competencies) to diagnose, treat, and prevent FGS. Establishing what health workers must know is a crucial first step towards the integration of FGS into women’s health care. The competencies are now available and can be used as a framework in the development of training for health professionals, opening the doors to better reproductive health for women.

**Supplementary Information:**

The online version contains supplementary material available at 10.1186/s12978-021-01252-2.

## Résumé

**Introduction:**

La bilharziose ou schistosomiase est une maladie aiguë et chronique causée par des parasites, qui peut prendre deux formes principales: intestinale ou urogénitale. Si elle n'est pas traitée, la forme urogénitale peut conduire à une bilharziose génitale féminine (BGF) entraînant fréquemment de graves complications en matière de santé reproductive chez les femmes et les jeunes filles. Les séquelles physiques causées par la BGF sont souvent mal diagnostiquées et fréquemment confondues avec celles causées par des infections sexuellement transmissibles (IST) ou des cancers du col de l'utérus. Malgré son impact sur la santé reproductive des femmes, la BGF est généralement négligée dans la formation médicale et reste méconnue, avec une faible sensibilisation à la fois dans les communautés touchées et chez les professionnels de la santé. La BGF a été décrite comme l'un des problèmes de santé sexuelle et reproductive les plus négligés en Afrique subsaharienne (Swai in BMC Infect Dis 6:134, 2006; Kukula in PLoS Negl Trop Dis 13:e0007207; Joint United Nations Programme on HIV/AIDS (UNAIDS) 2019). Une meilleure connaissance et une plus grande sensibilisation à la BGF sont nécessaires pour mettre fin à cette négligence, améliorer la santé reproductive des femmes et diminuer le fardeau de cette maladie tropicale négligée évitable et traitable.

**Méthode:**

Nous avons organisé des ateliers virtuels interactifs en collaboration avec l'Organisation mondiale de la santé (OMS), réunissant 64 participants du monde entier formés en médecine ou santé publique, afin d'établir des aptitudes (ou compétences) requises pour la prévention, le diagnostic et le traitement des BGF à tous les niveaux du système de santé. Les compétences ont été rédigées en petits groupes, révisées par des pairs et finalisées par les participants eux-mêmes.

**Résultats:**

Ce processus participatif a permis d'identifier 27 compétences nécessaires à la prévention, au diagnostic et au traitement de la BGF pour deux catégories de professionnels de santé: ceux travaillant dans un cadre clinique et ceux travaillant dans un cadre communautaire. Parmi celles qui concernent le milieu clinique, dix compétences sont liées au diagnostic de la BGF, dont trois impliquant un examen pelvien et sept n'en impliquant pas. Six se réfèrent aux comportements requis pour traiter adéquatement la BGF. Parmi les onze compétences qui concernent le milieu communautaire, six ont trait à l'identification des femmes à risque et cinq à la prévention.

**Conclusion:**

Définir les compétences nécessaires à la prise en charge de la BGF est une étape essentielle qui permettra de progresser vers un diagnostic et un traitement adéquat des femmes et des filles en Afrique subsaharienne par des professionnels de santé qualifiés. Les compétences suggérées peuvent dès à présent servir de base à la création d'outils éducatifs et de programmes d'enseignement pour mieux former les travailleurs de la santé à la prévention, au diagnostic et au traitement de la BGF.

## Resumo

**Contexto:**

A esquistossomose é uma doença aguda e crônica causada por vermes parasitas, que se apresenta de duas formas principais: intestinal ou urogenital. Se não for tratada, a forma urogenital pode resultar em mulheres e meninas à esquistossomose genital feminina (EGF); frequentemente resultando em graves complicações na saúde reprodutiva que são por vezes mal diagnosticadas como doenças sexualmente transmissíveis (DSTs) ou podem ser confundidas com câncer cervical. Apesar de seu impacto na saúde reprodutiva da mulher, a EGF é normalmente negligenciada no treinamento de saúde e permanece pouco reconhecida, com baixa conscientização tanto nas comunidades afetadas, como para os profissionais de saúde. A EGF tem sido descrita como uma das questões de saúde sexual e reprodutiva mais negligenciadas na África Subsaariana (Swai in BMC Infect Dis 6:134, 2006; Kukula in PLoS Negl Trop Dis 13:e0007207; Joint United Nations Programme on HIV/AIDS (UNAIDS) 2019). Um maior conhecimento e conscientização da EGF é necessário para acabar com esta negligência, melhorar a saúde reprodutiva das mulheres e diminuir o peso desta doença tropical negligenciada que é previnível e tratável.

**Metodologia:**

Realizamos workshops virtuais interativos, em colaboração com a Organização Mundial da Saúde (OMS), envolvendo 64 participantes com formação médica e de saúde pública de todo o mundo para estabelecer as habilidades (ou competências) padronizadas para prevenção, diagnóstico e tratamento de EGF em todos os níveis do sistema de saúde. As competências foram redigidas em pequenos grupos, revisadas por pares e concluídas pelos participantes.

**Resultados:**

Este processo participativo levou à identificação de 27 habilidades necessárias para a prevenção, diagnóstico e gestão da EGF para duas categorias de trabalhadores da saúde; aqueles que trabalham em um ambiente clínico, e aqueles que trabalham em um ambiente comunitário. Entre elas, dez estão relacionadas ao diagnóstico da EGF, incluindo três que envolvem um exame pélvico e sete que não o realizam. Seis constituem as condutas adequadas necessárias para tratar a EGF em um ambiente clínico. Onze tratam do ambiente comunitário, sendo seis relacionados à identificação de mulheres em risco e cinco relacionados à prevenção.

**Conclusão:**

Definir as habilidades necessárias para a administração da EGF é um passo crítico para proporcionar o diagnóstico e tratamento adequado de mulheres e meninas na África Subsaariana por profissionais de saúde treinados. As competências sugeridas podem agora servir como base para criar ferramentas educativas e currículos para melhor treinar os profissionais de saúde na prevenção, diagnóstico e gestão da EGF.

## Background

Schistosomiasis is an acute and chronic disease caused by parasitic worms, that can take two main forms: intestinal or urogenital [4]. According to the World Health Organization (WHO), in 2019 there were almost 240 million schistosomiasis infections worldwide, causing an estimated 3.3 million disability-adjusted life-years [2]. The number of deaths due to schistosomiasis is estimated between 24,067 and 200,000 globally each year, the highest mortality among the neglected tropical diseases recognized by the WHO [5]. Sub-Saharan African bears the majority of the global burden, reporting at least 93% of cases [6].

**Fig. 1:**
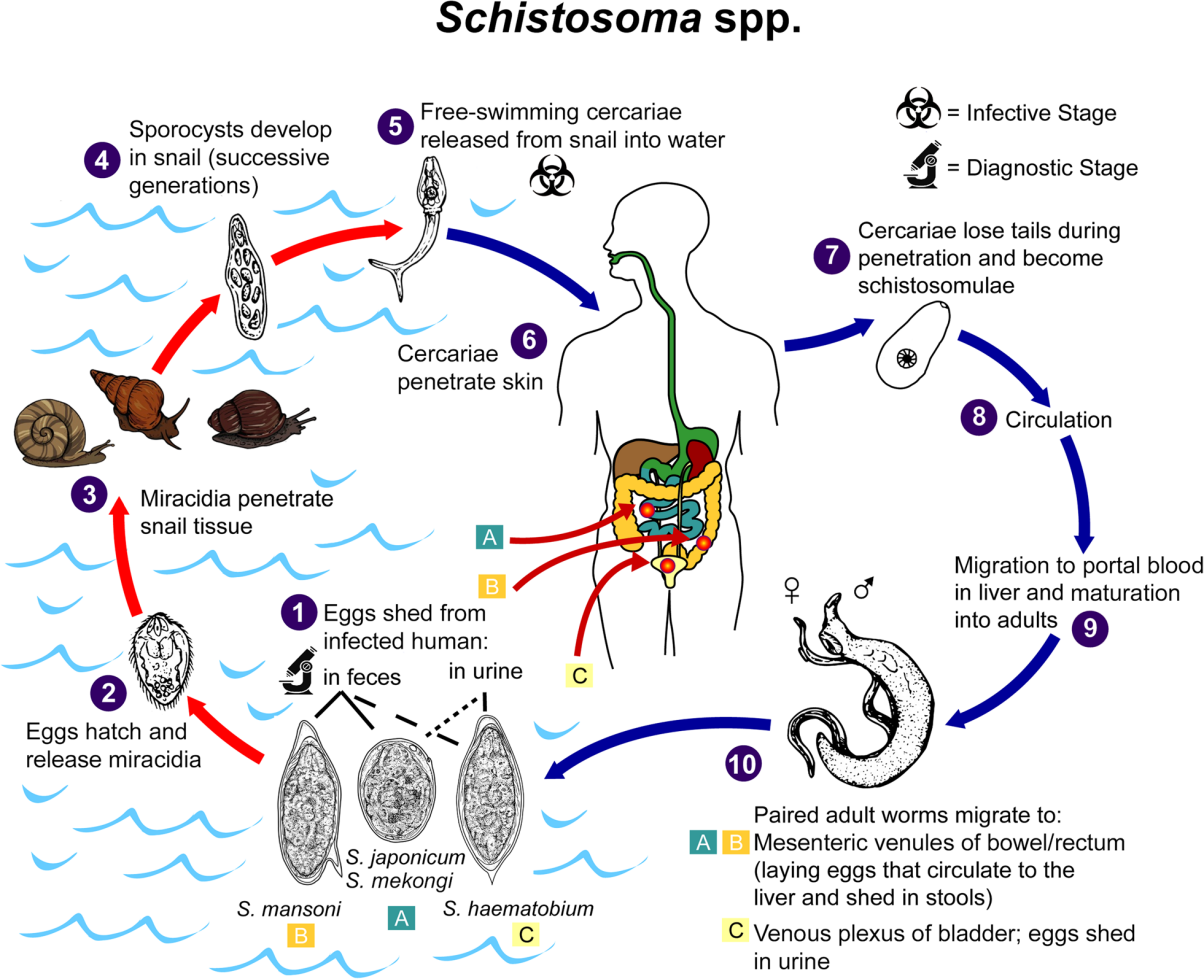
Transmission cycle of *Schistosoma* spp. Illustration depicting the life cycle of flatworms of the genus, Schistosoma, the causal agents of the parasitic disease schistosomiasis. A complete description of the *Schistosoma haematobium*, *S. intercalatum*, *S. japonicum*, *S. mansoni*, *S. mekongi* life cycle, is [7] available at: https://www.cdc.gov/dpdx/schistosomiasis/index.html. Figure provided by A. J. da Silva and M. Moser for copyright-free dissemination through the Public Health Image Library of the Centers for Disease Control and Prevention

Schistosomiasis is transmitted through contact with contaminated fresh water sources during normal daily activities, like washing, bathing, cooking, or swimming (Fig. 1) [6, 7]. Although there are several species of parasitic worms that can cause schistosomiasis, approximately two-thirds of cases are due to infection with *S. haematobium*, the primary cause of urogenital disease including female genital schistosomiasis (FGS). FGS is estimated to affect up to 56 million women and girls in Africa [8]. For example, a pilot study conducted in four rural communities in Ogun State in Nigeria showed schistosomiasis infection rates for women of up to 47% [9]. In Ghana, urinary schistosomiasis prevalence among women in the Volta basin was 24.8% while 10.6% of them were diagnosed with female genital schistosomiasis (FGS) [10].

Urogenital schistosomiasis affects both the urinary and genital track of infected individuals when eggs released by adult worms implant in tissue in the urogenital system of the infected host. The urinary signs and symptoms are easily recognized, with bloody urine often being the first sign of infection and typically what is described in medical training. Female genital symptoms include vaginal discharge, post-coital bleeding, genital burning, and/or pelvic pain [5]. If left untreated, infection can lead to more severe complications related to women’s reproductive health, characterized by anemia, sub- or infertility, spontaneous abortion, and ectopic pregnancy [11]. Community-based studies in two *S. haematobium*-endemic areas in Zimbabwe, reported that 15% of women suffered from infertility with an odds ratio (OR) of 3.6 for FGS [12]. A study analyzing DHS data in Ethiopia, Uganda, Kenya, and Tanzania demonstrated that women in *S. haematobium*-endemic areas had a significantly higher OR for infertility than those living in non-endemic areas suggesting that FGS may be an underlying factor [12]. A study conducted in Ghana, comparing 41 pregnant women infected with *S. haematobium* to 500 noninfected women, showed that the risk of premature birth was higher (34.8%) in infected women, compared to the others (23.8%) [13]. However, reliable and detailed data on the consequences of schistosomiasis and birth outcomes is still limited.

In addition to the pathology from the infection directly, FGS is a plausible risk factor for HIV acquisition [14–17] and cervical cancer [11, 18, 19] due to the tissue pathology, mucosal changes, and local immunologic modulations associated with FGS [20, 21]. In fact, studies across sub-Saharan Africa have shown a strong association between HIV prevalence and FGS [5]. For example, a case-controlled study in rural Zimbabwe showed that women with FGS had a three-fold risk of having HIV [17]. A study looking across 43 sub-Saharan countries found that for every *S. haematobium* infection per 100 individuals, there was a relative increase of 2.9% in HIV prevalence suggesting an association between infection with *S. haematobium* and HIV [14]. The local inflammatory response to the parasite eggs includes HIV target cells (CD4 + T lymphocytes and macrophages) [22] and coupled with the friable epithelium and lesions which can cause bleeding during coitus creates a permissive setting for HIV transmission [23]. Therefore, addressing schistosomiasis can improve women’s overall reproductive health and decrease vulnerability to other important reproductive health threats such as HIV. Recently, a WHO Technical Working Group on HIV and Schistosomiasis reviewed and summarized the evidence of the association between HIV and schistosomiasis and put forth concrete actions to control the HIV/schistosomiasis syndemic in adolescent girls and young women (AGYW) [3, 23]. In their systematic review [23] the authors, members of the working group, highlighted the need for training in health care workers as a critical gap to improve the condition of AGYW suffering from both diseases. The work we are presenting through this article, builds upon this evidence, underscoring the need for wider awareness on the disease’s characteristics, preventive measures, diagnostic and treatment amongst practitioners that care for the sexual and reproductive health of women and girls.

**Fig. 2:**
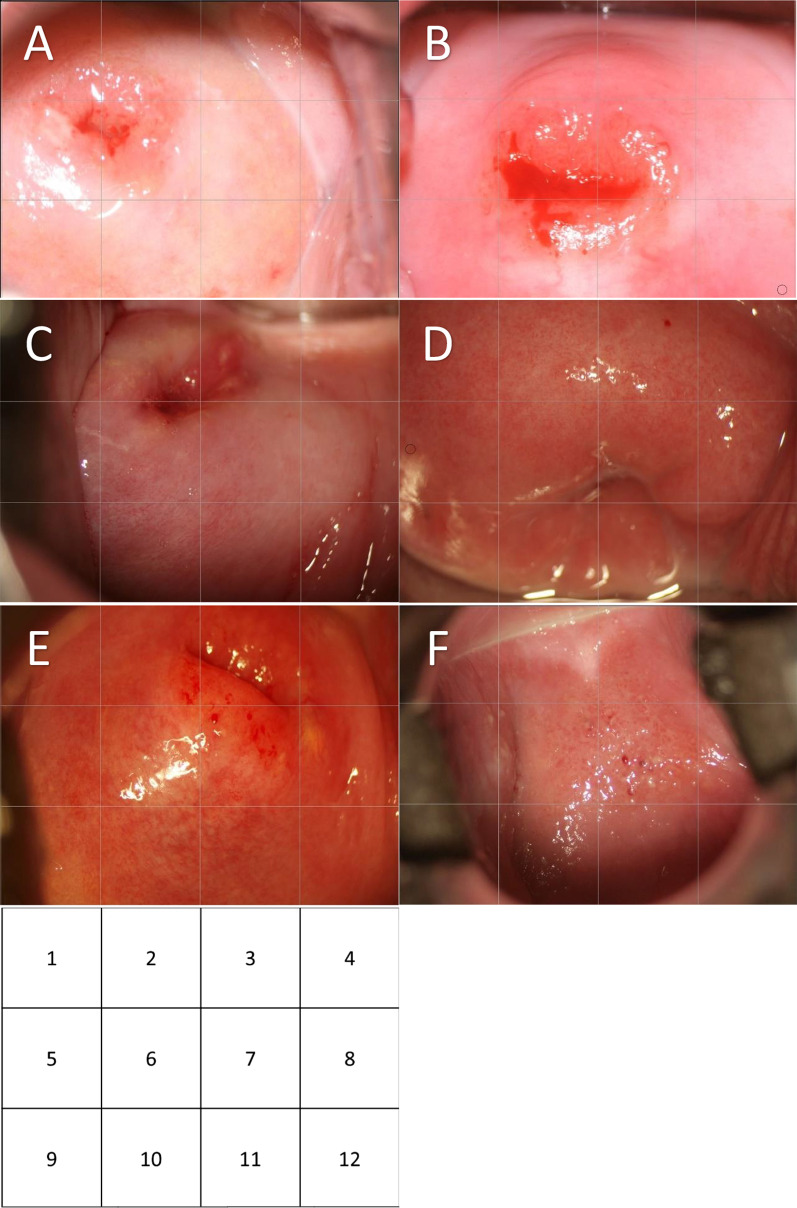
Title: Images of confirmed FGS cases. **A** Quadrants (Q) 3, 7, 9, 11 Homogenous yellow patches with grains interspersed (seen if image is full size. In Q 11 lower right corner abnormal blood vessels can be seen. Abnormal blood vessels can also be seen at the bottom of Q 10. **B** The classic circular abnormal blood vessels can clearly be seen at the bottom of Q 11 and 12. These circular features can also be seen more subtly in other quadrants. **C** Grainy sandy patches are seen in Q 2, and also in Q 6–8. Circular abnormal blood vessels are most clearly seen at the top of Q 10 but they are also in Q 6 and 11. **D** Abnormal blood vessels Quadrants 1–8. The size of the circular abnormal blood vessel is exemplified in Q 9. Single grains can be seen in Q 2 and 3. **E** Homogenous yellow sandy patches can be seen in Q 6–7 (and the near in the quadrants around). Circular and uneven-calibered blood vessels are sprawled across this area and there is contact bleeding Q2 and Q7. A Nabothian follicle can be seen in Q 8, here the blood vessels are normal, straighter, evenly branching across the follicle. **F** Rubbery papules are seen in Q 5–6 on the anterior surface of the cervix. The anterior fornix is seen at the top of Q 2. **G** The quadrants. Images were captured by Elisabeth Kleppa and Eyrun Kjetland

Many non-specific symptoms such as vaginal discharge and pain that patients present with result in many cases being misdiagnosed as sexuality transmitted infections (STI). The diagnosis of FGS is made clinically through colposcopy or visual inspection identifying lesions on the cervix or vaginal tissue [24]. Lesions are described in the WHO FGS Atlas [25] and include intra-vaginal classic grainy sandy patches, single or clustered grains, homogenous yellow patches, rubbery papules, and abnormal blood vessels as seen in the images in Fig. 2 [25]. In addition, some case reports show hypertrophic or ulcerative lesions on the vagina, vulva, or cervix [26–28]. These lesions have been linked histopathologically, in some cases, to the presence of eggs in the tissue causing inflammatory reactions. Detection of these lesions requires colposcopy, or for large lesions, via visual inspection with a speculum or biopsy. However, colposcopes are not commonly available in most rural endemic settings and therefore most infected individuals are never diagnosed. Even if women are referred to higher levels in the health system, the lesions are often not identified as FGS and again proper treatment is not provided.

When an FGS case is suspected, the appropriate treatment of Schistosoma infection is with 40 mg/kg of praziquantel as a single dose based weight or according to local guidelines with repeated treatment if risk factors persist. Treatment for school-aged children is donated by Merck kGaA, Darmstadt, Germany, and freely available as a community-based treatment regardless of infection status in a process known as mass drug (or medicine) administration (MDA). As infection in endemic settings is frequently asymptomatic until parasite loads increase and tissue damage has progressed, prevalence at the community level in school-aged children will trigger MDA to reduce morbidity and progression of disease in the population. Individual patient treatment for FGS is also with single dose treatment although clinical trials are ongoing to see if different treatment regimens are more effective. Treatment is most effective when given early; however, the potential for reinfection remains high without improved access to safe water [29]. Lesions found later in life may not resolve with antiparasitic treatments as some eggs remain in tissues despite the death of the adult worms and because of fibrosis and calcification of the lesions. More studies are needed on the treatment of advanced disease.

**Fig. 3:**
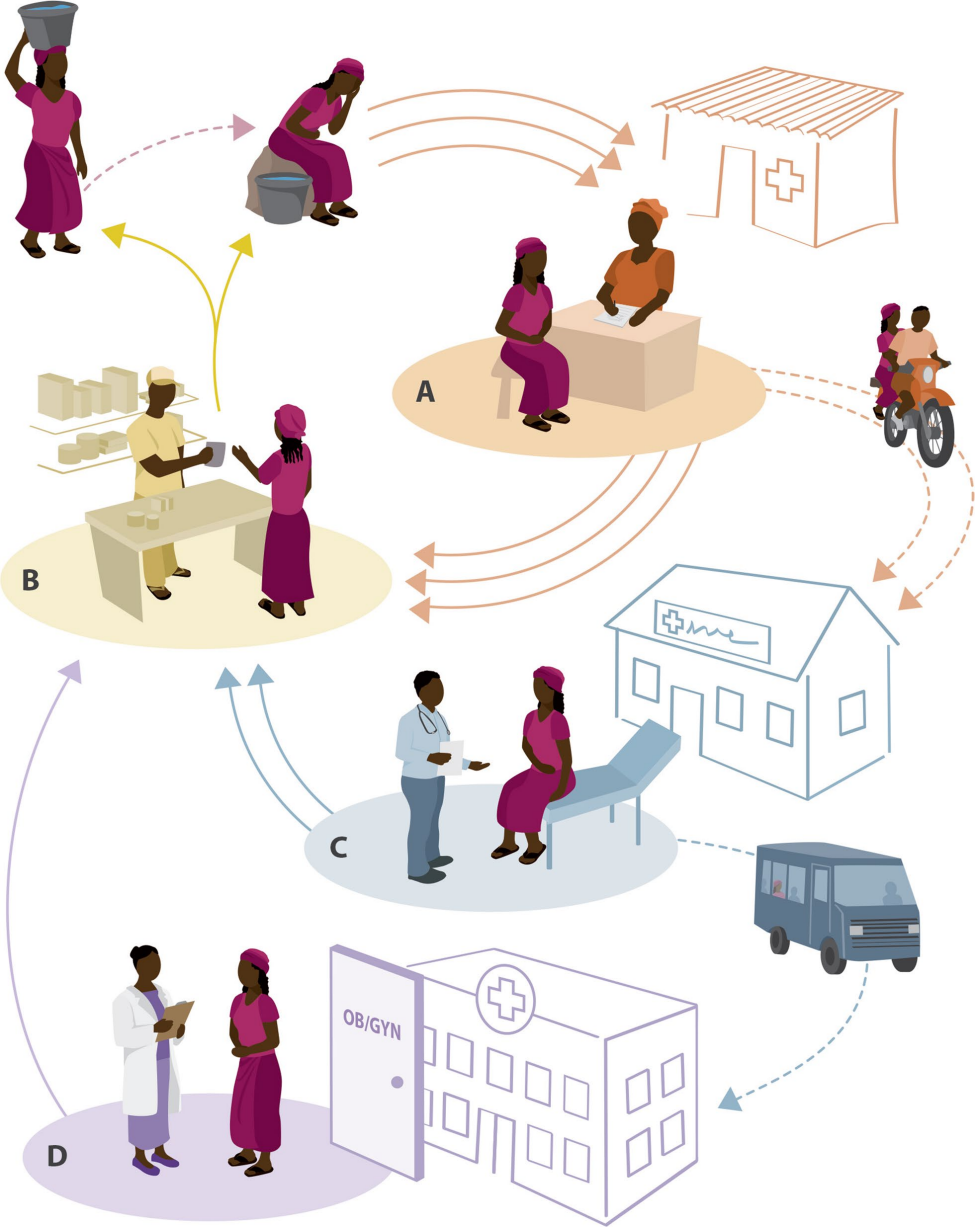
Cycles of FGS Misdiagnosis and Treatment. FGS afflicted woman presents to local health post (**A**) and leaves to receive treatment at the local pharmacy (**B**) where she most likely is treated for STI which leaves her with symptoms and this same cycle may be repeated. Without resolution the woman may be referred to the next level health center (**C**) where again she is most likely treated for STI (**B**), again without resolution. The woman may be referred to the next level health center (**D**) where further exam and tests are done however, she again is most likely treated for STI (**B**) and does not return to her healthy state. With FGS diagnosis at any facility (**A**, **C**, or **D**) the woman could be treated with praziquantel and returned to a healthy state

Recognition and proper treatment of FGS is greatly hampered by the fact that it may not be part of standard medical training. In addition, awareness of the disease is low in both affected communities and health systems of endemic countries leaving women little recourse to address the symptoms and complications associated with FGS [2]. Figure 3 shows the most common cycles of FGS misdiagnosis and treatment. In cycle A, a woman is exposed to contaminated water through activities of daily living and becomes ill. She most commonly seeks care at the local health post where, based on symptoms that likely including vaginal discharge and pain, she is presumptively treated for sexually transmitted infections (STI). The woman would then go to the local pharmacy in the village and get antibiotic treatment for STI. When symptoms do not resolve, treatment may be repeated with non-compliance or re-infection being suspected. Eventual referral to the next level health facility would typically require transport and a longer time away from home and household or work responsibilities. At the next level facility depicted in cycle C, there would likely be a further exam but with a similar outcome, perhaps treating with next-line therapy for STI. The woman would return home and again receive antibiotic treatment and again symptoms would not resolve. This process could be repeated with cultures taken and while awaiting results repeating treatment. Without resolution, the patient would then be potentially referred to the next level facility in cycle D. Upon referral the patient would receive a pelvic exam and cultures and presumptive treatment may commonly be repeated again. If a pelvic exam is done and a lesion visualized, biopsies may be taken which again would result in treatment delays and potentially more aggressive treatment if presumptive diagnosis of cervical cancer is suspected. Every visit, treatment, and referral point in this cycle has financial and opportunity costs for the woman and is a potential loss to follow up. Each heath care visit also represents an opportunity to break this cycle if FGS is suspected, providing the opportunity to go from the pharmacy with the correct treatment and return to an improved state of health. This image also shows the potential role that the pharmacy can play as a common point in the referral cycle. These cycles of misdiagnosis and mistreatment demonstrate the need to increase awareness and knowledge around detection and proper treatment of FGS at all levels of the health care system so women and girls no longer have to suffer the consequences of inadvertent misdiagnosis and improper treatment. As a first step, a consistent framework is needed for training health professionals on how to diagnose, treat, and prevent FGS.

As part of the FGS Accelerated Scale Together (FAST) Package project and in collaboration with the WHO NTD department, Bridges to Development and the Geneva Learning Foundation conducted a virtual interactive workshop to establish the competencies that are required train health workers in FGS at various levels of the health systems in endemic settings. The objective of this work was to create a durable and openly available resource that any organization, health center, or medical institution can use to facilitate the integration of FGS training into their programs or curricula. We believe that increased awareness of and training for FGS will ultimately help endemic countries address the needs of AGYW at risk of FGS within their health systems.

## Methods

Competencies for FGS are defined as skills, knowledge, and actions required to diagnose, prevent, and treat FGS. The competencies necessary for the management of FGS were developed through a virtual workshop consisting of four sessions taking place over the course of three weeks in July and August 2020. The workshop was hosted by Bridges to Development in collaboration with the WHO and facilitated by the Geneva Learning Foundation (GPLF). The workshop is part of the FAST Package, a project funded by Grand Challenges Canada and the Government of Canada, and was sponsored by the Coalition for Operational Research on Neglected Tropical Diseases (COR-NTD) with funding from the Foreign, Commonwealth, and Development Office (FCDO) of the UK government. The objectives, approach, and proposed outputs of the workshop were developed by a small planning group consisting of members of the WHO NTD Department, select physicians and researchers working in FGS (Table 1). A key goal for the workshop was to engage a diverse group of participants both geographically and professionally to aim for a comprehensive output generalizable to different contexts. The initial list of participants was built upon the networks of the members of the planning group and the WHO team to strategically reach out to appropriate individuals with relevant experience in sexual and reproductive health, health education, parasitic diseases, NTD programs, and public health.

**Table 1:** Composition of FGS Competencies Workshop Planning Group

Name	Institution
Alain Blaise Tatsinkou	The Geneva Learning Foundation
Amadou Garba Djirmay	Word Health Organization
Anastasia Pantelias	Bridges to Development
Clara Fabienne Rasoamanamihaja	Ministry of Health Madagascar
Daniela Fusco	Bernhard Nocht Institute for Tropical Medicine
Elisabeth Long	Coalition for Operational Research on NTDs
Eyrun Kjetland	University of KwaZulu-Natal, South Africa/ Oslo University Hospital, Norway/ BRIGHT Academy, Ugu District, South Africa
Julie Jacobson	Bridges to Development
Kayla Hendrickson	Bridges to Development
Kazeem Adebowale Adekunle	University of Sierra Leone teaching hospital complex connaught
Mbolatiana Raharinivo	Ministry of Health Madagascar
Megan Williamson	Bridges to Development
Olabanji Surakat	Osun state University, Nigeria
Pamela Sabina Mbabazi	Word Health Organization
Reda Sadki	The Geneva Learning Foundation

The workshop was divided into four virtual sessions: an introductory session (session 1); two working sessions (sessions 2a and 2b); and a final review session (session 3). The first, two-hour introductory session included presentations on FGS and the purpose and objectives of the workshop. Also included was an orientation to the highly interactive competency-development and peer-review process to be used in the following working sessions. The second session, which was hosted in both English and French, was divided into two parts to account for the different roles of health professionals and tools available at different levels of the health system. Working session 2a focused on health professionals who work in a clinical setting and who are able to perform pelvic exams either by colposcope or only speculum. Participants in this session included OB/GYNs, physicians, and nurses. Working session 2b focused on health professionals who work in a community setting or health clinic who are not able to perform pelvic exams. Participants in this session included community health workers, researchers, parasitologists, pharmacists, and public health practitioners. Lastly, to better focus the workshop, competencies were developed against the five most critical behaviors related to FGS; identify women at risk, prevent, refer, diagnose, and treat. Competencies related to diagnosis of FGS using radiological methods were beyond the scope of this workshop as most surface lesions are too small for radiology [30]. Routine skills in sexual and reproductive health like detection and care for STI, HIV, or cervical cancer, how to take a general history or perform a pelvic exam were beyond the scope of this workshop.

During the two working sessions, participants worked in small groups to develop draft competencies using a template (Table 2) to ensure consistency in structure and elements across each group. Each competency had to answer the following questions: “who, does what, to whom, for what, when, and requiring what background knowledge”. Each group’s draft competencies were then subject to peer-review. To facilitate the peer-review process, the participants used a collaborative platform called *PeerGrade* where, using a check list (review rubric), they were prompted to provide concrete, specific, and actionable feedback. At the end of each working session, peer-review feedback was compiled and used to revise the draft competencies.

**Table 2:** Template for drafting FGS competencies

Which group are you in?
Heath worker who performs pelvic exams
Heath worker who does not perform pelvic exams
Which category are you addressing?
Diagnose FGS in a clinical setting
Perform pelvic exam to clinically diagnose FGS
Treat schistosomiasis and FGS
Identify women at risk in the community
Prevent schistosomiasis and FGS
Draft the competency written in ABCD format (Audience + Behavior + Condition + Degree) answering the below questions:
Who? (Audience)
Does what? (Behavior)
When/how? (Condition)
To what degree in order for it to be correct? (Degree)
Is a specific level of expertise or experience useful or required for this competency?
Is there any additional context needed to understand this competency?

The final review session allowed participants to review the revised draft competencies and provide final feedback. Again, this work was performed in small groups to benefit from the expertise and experience of all participants. The goal of this work was to ensure that the competencies were clear, relevant and generalizable across participants’ countries and contexts. As a final step, the competencies were shared with the WHO NTD program for final review. This step ensured the outputs were aligned with WHO guidelines and the WHO FGS Atlas [25] and that there were no significant gaps. The workshop was evaluated by participants.

## Results

**Fig. 4:**
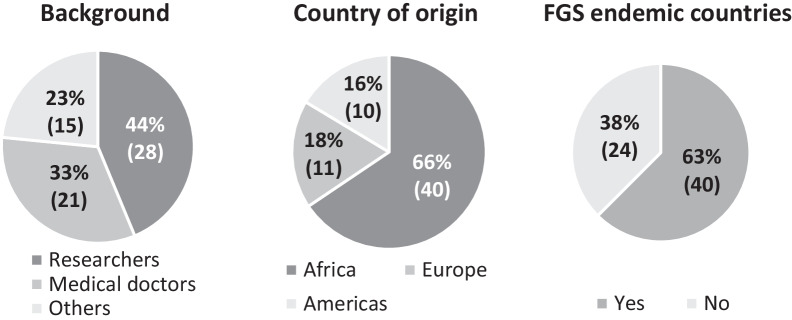
Breakdown of Workshop participants

**Fig. 5:**
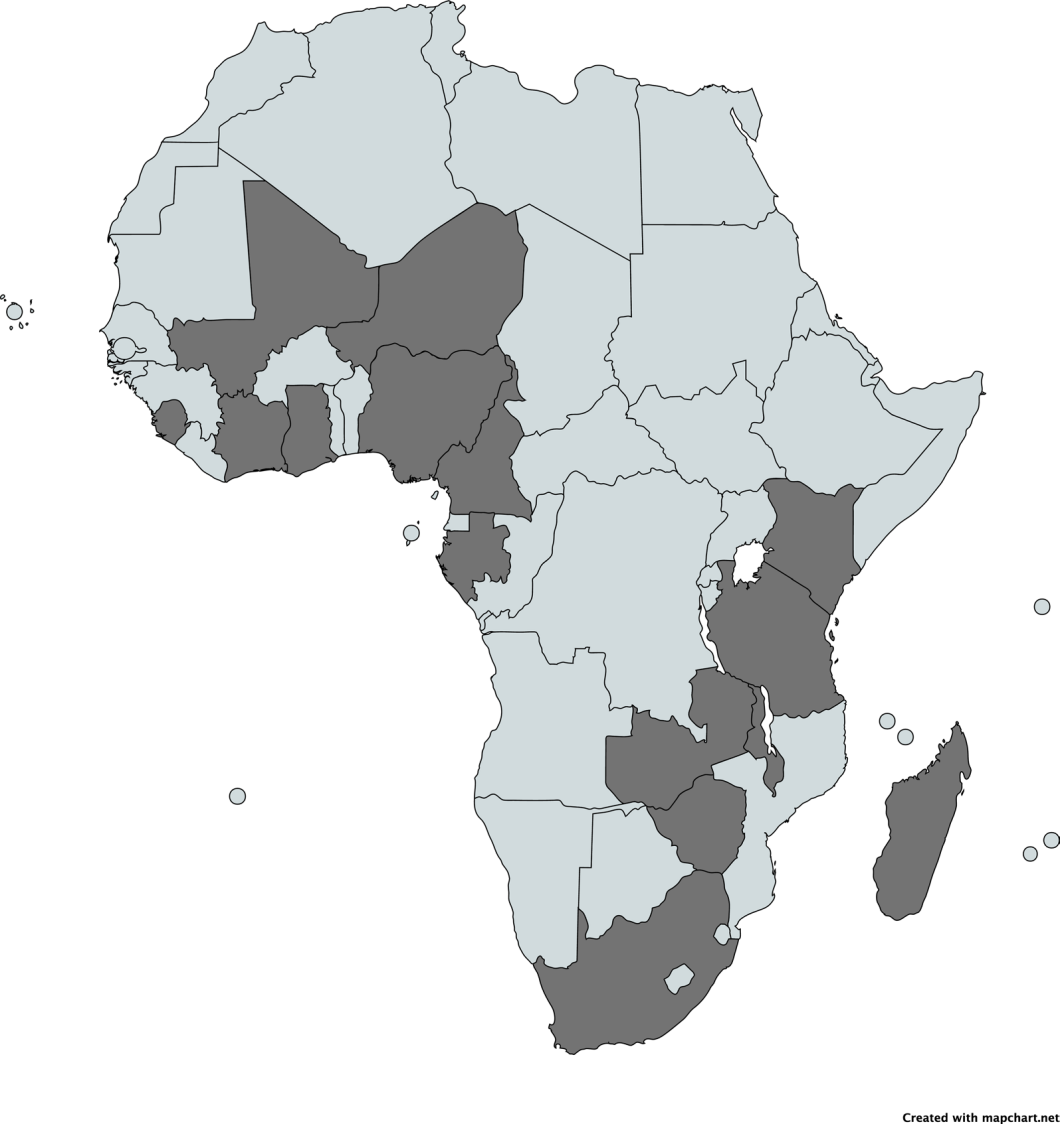
Map showing countries in which participants reported doing FGS-related work

The competencies were developed by 64 participants, consisting of researchers [28], medical doctors [21] and other professions [15] with FGS-related projects, coming from 24 different countries (Fig. 4). Some of the participants had prior experience as nurses or midwives. Most of the “other professions” were pharmacists, parasitologists, or support staff for HIV community programs. The workshop included a high representation of endemic countries with 16 endemic Francophone and Anglophone countries participating (Fig. 5) which helped with the aim of creating competencies which are as comprehensive and generalizable to endemic areas as possible.

**Fig. 6:**
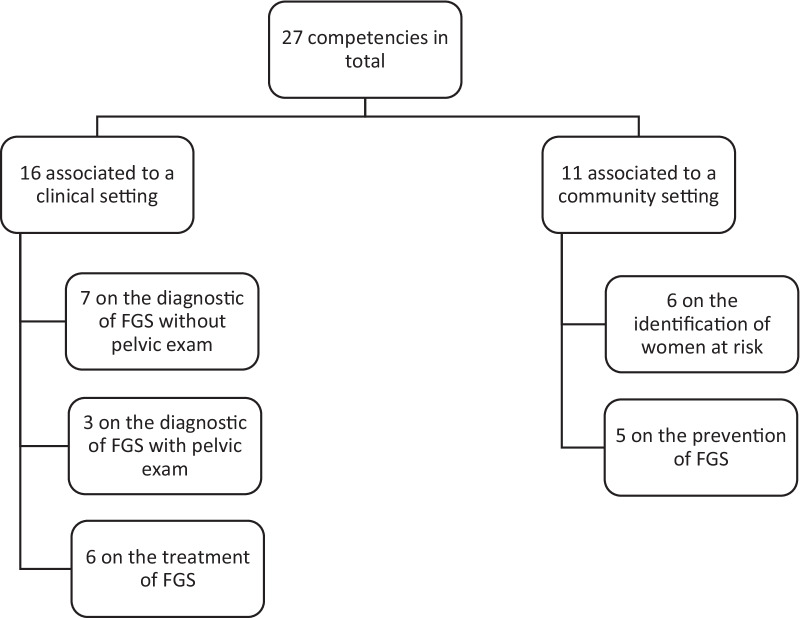
Competencies, categories and sub-categories

The two interactive workshop sessions (2a and 2b) resulted in approximately 65 draft competencies. After consolidation taking into account the feedback collected on *PeerGrade,* a set of 31 draft competencies were developed reflecting the input of all participants. Workshop participants discussed and revised this set further prior to submitting to the WHO NTD Program for final comment and approval. As a result of this process, the final output of the workshop included 27 competencies across two categories of health workers: those working in a clinical setting, and those working in a community setting (Fig. 6). The competencies are designed to cover the different steps in caring for an at-risk individual from identifying risk factors, taking of relevant history, counseling on risk for HIV acquisition, and presumptive treatment for FGS. The two-category breakdown reflects the differences in technical resources and knowledge available in these two types of settings. For example, the community competencies are focused on the interactions between a health worker and community members in FGS-endemic areas. Competencies included focus on the ability to ask appropriate questions about symptoms, risk factors, and exposure history to identify those at high risk for FGS. Also included are competencies focused on the ability to deliver information on the disease and how women can access treatment, counsel them on appropriate behaviors to prevent FGS and correctly administer praziquantel to treat women with suspected or confirmed FGS.

The clinical setting competencies address a more purely medical and technical approach in addition to the identification and counseling components described above and focus on the unique attributes of FGS within the context of health seeking for sexual and reproductive health care. For example, the competencies cover behaviors such as diagnosing FGS using a speculum to inspect the women’s cervical and all vaginal surfaces for lesions. Included are competencies focused on ordering laboratory tests or performing visual inspection with acetic acid/visual inspection with lugol’s iodine (VIA/VILI) to differentiate FGS from STIs or cervical cancer lesions respectfully. Overall, this set of competencies are focused on schistosoma-specific issues and prevention within the context of community contacts for multiple services including sexual and reproductive health needs including HIV and cervical cancer prevention. The detailed description of the final set of 27 competencies can be found in this article's supplementary materials (Additional file 1).

Despite taking place virtually, in a post-workshop survey, 89% of participants reported learning more about FGS and 100% reported that the workshop increased their motivation and commitment to the work. Another potential outcome of the workshop is a new FGS Community of Practice as 92% of participants stated they would like to remain connected moving forward. This increased awareness and motivation around FGS is critical to meaningfully address the burden of this disease. Overall, the results of this work are the first ever comprehensive description of the skills required for health workers, at all levels, to be able to prevent, diagnose, treat, and identify women and girls at risk for FGS. These results can then be used as the basis for training and evaluating health workers on FGS.

## Discussion

To address the burden of FGS across sub-Saharan Africa, it is essential that FGS care and treatment is integrated into routine sexual and reproductive health services where women and girls affected by the parasite will most likely seek care. The workshop identified items that can henceforth be used for training health professionals in the management of FGS. Clinicians should be taught how to identify FGS during pelvic exams, what to say to the patient and how to manage the disease. Other health workers should be trained in informing women about FGS and the importance of early preventive chemotherapy.

Due to lack of awareness both in endemic communities and the health care system, FGS remains one of the most important misunderstood areas in women’s and girl’s health. Lack of knowledge, expertise, and training in health care providers, has resulted in under or misdiagnosis of FGS, limiting access to treatment for women and girls suffering from this preventable and treatable disease. Dr Tedros Adhanom Ghebreyesus, Director-General, World Health Organization, has stated that “FGS is a silent and neglected epidemic, affecting the same people who carry a disproportionate global burden of HIV and cervical cancer” [3]. The reproductive health consequences such as infertility, sub-fertility, miscarriage, and ectopic pregnancy are often accompanied by critical social consequences such as discrimination and stigma, particularly when FGS is mistaken for an STI [2, 31]. Therefore, improving health care workers’ knowledge by incorporating FGS-related material into medical training curricula can help address this awareness gap and improve women’s reproductive health. The first step towards this goal is to clearly define a standardized set of learning outcomes; specifically, the competencies or behaviors required to adequately prevent, diagnose, and manage an FGS case.

The definition of these competencies is a crucial step towards the appropriate integration of FGS into women’s health care. The availability of the FGS competencies can open the door for the development of new, comprehensive training curricula for all health professionals who may contact women and girls at-risk or suffering from FGS. These competencies are a global good, meant to provide the foundation for any program seeking to develop training for FGS.

One of the strengths of the virtual setting for the workshop and the methods used to develop the competencies was the ability to gather input from a highly diverse set of participants and to foster a deeper understanding and awareness of FGS. The diversity of participation was also important for developing competencies which adequately reflect the context in which FGS occurs. For example, as a neglected tropical disease, FGS mainly affects people living in remote areas with limited resources. Health care facilities in endemic areas may not have the tools and interventions needed to diagnose and treat FGS such as colposcopy or praziquantel. Additionally, because FGS is often confused with STIs, affected women and girls often suffer from stigma affecting their ability to access appropriate care [2]. Therefore, particular attention was paid to the different context and environments where FGS cases occur taking into consideration the different health infrastructures, resources and healthcare workers capacities in FGS-endemic countries and communities. This inclusive approach and the diversity of the participants panel resulted in competencies that can likely be adapted and tailored to the environment and settings in which they are being used and the diagnosis of presumed FGS versus clinically confirmed FGS [20].

A limitation of this work is that training of medical professionals alone, while required, is not sufficient to fully address the problem of FGS. Training for FGS needs to be part of a holistic approach to prevention, diagnosis, and treatment which considers the needs at an individual, community, and health-system level. A holistic approach, in addition to training of medical professionals, would include activities such as: (1) building awareness of FGS, associated risk-factors, and reproductive health sequelae in at-risk communities and schools [19]; (2) promoting and ensuring uptake of annual mass drug administration with praziquantel in all schistosomiasis-endemic communities and schools [20, 21]; and (3) integrating diagnosis and treatment of FGS in current algorithms for STIs and cervical cancer and ensuring cases are reported and integrated into the routine health management information system [3]. This holistic approach can help decrease the burden of FGS by both preventing new cases and ensuring improved access to appropriate care for women and girls suffering from FGS. This holistic approach will be piloted in Ghana and Madagascar in 2021 under the FAST Package project.

As a next step in this project, new, interactive, digital training modules will be developed against these competencies and piloted with health workers in both Ghana and Madagascar as part of the FAST Package. Further training materials will need to be developed by country programs or their partners and within medical training programs in countries to build on this. To date there are not to our knowledge any standardized training packages available. Several small-scale projects are underway, several being conducted by workshop participants, and these competencies will help, ensure that there is comparability across training outcomes. Continued collaboration across workshop participants and others putting these competencies into curricula will further strengthen programs and allow women and girls to access the services and treatments they need.

## Conclusion

The significant lack of awareness and knowledge from health professionals around FGS has been an obstacle to the improvement of women’s reproductive health in many parts of sub-Saharan Africa where schistosomiasis is endemic. The prevalence of the disease in endemic countries can reach very high rates. However, training on FGS remains absent from medical curricula and health worker training materials in most countries. This knowledge gap results in the underdiagnosis, misdiagnosis, and/or lack of or inappropriate treatment for FGS contributing to poor reproductive health outcomes in endemic areas. Incorporating FGS-specific material into the training and education of health professionals can be a highly effective means to improve the prevention, diagnosis and management of FGS cases and consequently improve women’s reproductive health and contribute to the advancement of the sexual reproductive health rights agenda for women in Africa. Defining the competencies, to adequately diagnose, treat, and prevent FGS is a necessary first step towards this goal that can be used as a foundation to create new educative tools and materials on FGS for health professionals. By filling this knowledge gap, we hope to see FGS care and prevention integrated seamlessly into sexual and reproductive health services so the needs of this neglected population can be met and the suffering relieved.

## Supplementary Information

**Additional file 1.** FGS Competencies Table.

## Data Availability

Data and materials are available on request.

## Abbreviations

AGYW: Adolescent girls and young women
COR-NTD: Coalition for Operational Research in Neglected Tropical Diseases
FAST: FGS Accelerated Scale Together
FCDO: Foreign, Commonwealth, and Development Office
FGS: Female genital schistosomiasis
HIV: Human immunodeficiency virus
MDA: Mass drug (or medicines) administration
NTD: Neglected tropical diseases
OR: Odds ratio
VIA/VILI: Visual inspection with acetic acid/visual inspection with lugol’s iodine
WHO: World Health Organization
