# Supplementary material for: Addressing a silent and neglected scourge in sexual and reproductive health in Sub-Saharan Africa by development of training competencies to improve prevention, diagnosis, and treatment of female genital schistosomiasis (FGS) for health workers
Source: Reprod Health. 2022 Jan 24;19:20. doi: 10.1186/s12978-021-01252-2 (PMC8785555; doi:10.1186/s12978-021-01252-2)
Supplement: Supplementary file 1 — Additional file 1. FGS Competencies Table. [file 12978_2021_1252_MOESM1_ESM.pdf]

1 Competencies for medical professionals working in a clinical setting<br><br>1.1 Diagnose FGS in a clinical setting						
**#**	**Who**	**Does what**	**To Whom**	**For what**	**When**	**Required Knowledge**
**1.1a**	Health professional	Asks about the history/ lifetime risk factors of women and girls that live, have lived, or have traveled in an endemic area	Women and girls who come to health facilities	Determine potential exposure to schistosomiasis and risk of FGS	At every clinical visit by women and girls when appropriate.	Understand and recall the risk factors for FGS and know to ask family or those accompanying younger girls as appropriate for better history of potential risk
**1.1b**	Health professional	Asks appropriate questions about FGS symptoms (vaginal discharge, urinary incontinence, blood in urine, irregular vaginal bleeding, postcoital bleeding, lower abdominal pain, infertility), and previous treatment history and outcomes in routine clinical practice	Women and girls potentially at risk of schistosomiasis who come to health facilities	Determine if women and girls are experiencing symptoms consistent with FGS or urinary schistosomiasis (bilharzia)	At every clinical visit by women and girls when appropriate.	Understand and recall the symptoms of FGS as described in the WHO FGS Atlas, and recognize that failed treatment for STI increases suspicion of FGS
**1.1c**	Health professional	Asks about history and symptoms per 1.1a and 1,1b above	Women and girls that are referred with radiological or histopathological signs	Determine where schistosomiasis was acquired and who else may be at risk and require follow up	When patients referred	Understand risk factors and symptoms and radiographic and histopathologic signs of FGS

**1.1d**	Health professional	Elicits history from patient about previous treatment for sexually transmitted infections, infertility, screening for cervical cancer, and previous vaccination for HPV.	Women who present with blood in urine, clinical symptoms of FGS, or are from an at-risk community/exposure	Differentiate different diseases that may have similar signs and/or symptoms	Women present to clinic with blood in urine, clinical symptoms of FGS, or are from an at-risk community or are at risk of exposure	Aware that failed treatment for STIs or cervical cancer can be a sign of FGS
**1.1e**	Health professional	Orders laboratory diagnostic test for schistosome and STIs per local guidelines	Women and girls at-risk of FGS with bloody urine, clinical FGS symptoms, and/or a suspected STI not responding to treatment	Diagnosis of active parasitic infection and differentiate from STI	At clinical visit by women and girls when medical practitioner deems it appropriate.	Understand that even a negative UGS test does not exclude the diagnosis of FGS and praziquantel treatment is still warranted. Know and follow the local guidelines for diagnosis and treatment of STI.
**1.1f**	Health professional	Records suspected and confirmed FGS cases and links to care	Any woman or girl with presumed, suspected, or confirmed FGS diagnosis	Ensure documentation and reporting of FGS	Any clinical visit	Understand FGS case definitions, recording, and reporting process
**1.1g**	Health professional	Refer or confer with a local experienced colleague when in doubt of clinical findings, unable to perform pelvic exam or get adequate visualization in current setting, unclear on treatment, or when presented with a symptomatic patient but	Women and girls (including young or pre-sexually active women/girls) at-risk or with symptoms consistent with pelvic abnormality but unclear diagnosis	Ensure accurate diagnosis	During clinical visit or following visual inspection where diagnosis is not clear	Understand limitations of knowledge and understanding and resources available to utilize in assisting with diagnosis and management. Knowledge to

#	Who	Does what	To Whom	For what	When	Required Knowledge
		pelvic exam is not prudent per local norms and standards	following visual inspection			administer stat dose of praziquantel.
**1.2 Perform pelvic exam to clinically Diagnose FGS**						
**#**	**Who**	**Does what**	**To Whom**	**For what**	**When**	**Required Knowledge**
**1.2a**	Health professional who performs pelvic exams with visual inspection with or without colposcope	Uses a speculum with adequate lighting to inspect every vaginal surface (turn the speculum ninety degrees to inspect the lateral vaginal surfaces) and cervix to identify any lesions and to assess the color, size, and shape	Sexually active women and girls at-risk or with symptoms consistent with pelvic abnormality	Identify signs of FGS: grainy sandy patches, homogenous yellow patches, rubbery papules, abnormal blood vessels	At clinical visit by women and girls when medical practitioner deems it appropriate	Recognize FGS signs as contained in WHO Atlas during pelvic exam, when using colposcope or speculum
**1.2b**	Health professional who performs pelvic exams with visual inspection with or without colposcope	Performs the VIA/VILI procedure and report the local changes of appearance of the lesions and biopsy if indicated.	Sexually active women and girls at-risk or with symptoms consistent with pelvic abnormality	Differentiate cervical cancer lesions from other lesions such as FGS lesions	During clinical visit of women and girls with suspicious lesion on visual inspection or during cervical cancer screening.	Knows how to perform VIA/VILI and recognize lesions at-risk for cervical cancer. Note that lesions that are not shiny white (presentation of cervical cancer) and outside of the transformation zone increase suspicion of FGS
**1.2c**	Health professional who performs pelvic exams	Identifies the presence of discharge/ history of discharge – smell and color – and treatment for STI's	Women presenting with blood in urine, clinical symptom of	Differentiate STI that may have similar signs and/or symptoms and	Women present to clinic with blood in urine, clinical symptom of FGS, or from at-risk	Experience with the current treatment of STI

	with visual inspection with or without colposcope	according to local algorithms / management	FGS, or from at-risk community/exposure	ensure proper treatment	community/ exposure	
**1.3 Treat schistosomiasis and FGS**						
**#**	**Who**	**Does what**	**To Whom**	**For what**	**When**	**Required Knowledge**
**1.3a**	Health professional	Prescribes or administers 40mg/kg of praziquantel as a single dose based on height or weight dosing according to local guidelines and repeats treatment if risk factors persist	A patient diagnosed with FGS or presenting signs and symptoms indicative of FGS (suspected case) or living in or having traveled to an endemic area	To treat schistosomiasis	Whenever there is a confirmed, suspected, or exposed woman or girl	Understands appropriate treatment for schistosomiasis and FGS, the need for repeated treatment if risk factors persist, and is following current protocols
**1.3b**	Health professional	Manages or refers for treatment complications of FGS per the WHO FGS Atlas	A patient suspected or diagnosed with FGS with identified complications	To treat complications	Whenever there is a confirmed, suspected, or exposed woman or girl	Is familiar with the possible complications of FGS and adequate treatment or referral for further treatment
**1.3c**	Health professional	Performs or refers the patient for biopsy or treats according to treatment guidelines if there are aceto-white changes or any suspicion of cancer	A patient suspected or diagnosed with FGS in patient with suspicious lesions for cervical cancer	To prevent or treat cervical cancer	Whenever there is a confirmed, suspected, or exposed woman or girl	Is familiar with diagnosis and management of cervical cancer
**1.3d**	Health professional	Informs and counsels the patient about chronicity of established lesions and the higher risk for HIV acquisition if exposed to HIV	A patient suspected or diagnosed with FGS	To prevent on HIV and other STI	Whenever there is a confirmed, suspected, or exposed woman or girl	Understands appropriate treatment for schistosomiasis according to local algorithm and is following current

#	Who	Does what	To Whom	For what	When	Required Knowledge
						protocols for HIV prevention including pre-/post-test counselling
**1.3e**	Health professional	Informs and counsels the patient that family and community members may also be at risk for schistosomiasis and should seek treatment	A patient suspected or diagnosed with schistosomiasis or FGS	To prevent and treat schistosomiasis	Whenever there is a confirmed, suspected, or exposed woman or girl	Understands risk factors and epidemiology of schistosomiasis and its clinical complications
**1.3f**	Health professional	Presumptively treats patient with 40mg/kg of praziquantel as a single dose, based on height or weight according to local guidelines	Women who are presenting with infertility or sub-fertility from an area at risk	To treat schistosomiasis	Whenever there is a confirmed, suspected, or exposed woman or girl	Understands appropriate treatment for schistosomiasis and FGS. Understands that treatment may need to be repeated or administered regularly for individuals living in endemic areas with poor water & sanitation facilities
**2 Competencies for medical professionals in community settings**<br>**2.1 Identify Women at Risk in the Community**						
**#**	**Who**	**Does what**	**To Whom**	**For what**	**When**	**Required Knowledge**
**2.1a**	Community health worker	List communities that are either endemic for schistosomiasis or close to fresh water sources in endemic areas with which	Communities	To identify communities at risk for schistosomiasis	Prior to implementing interventions / action within the community or	Ability to access and use local health facility data, has knowledge of the disease in the area, and data

		community-members may have contact			during micro-planning exercises	(epidemiological) from the NTD program at national and sub-national level
**2.1b**	Community health worker/peer educator	Assess risk for schistosomiasis infection including questions about routine activities that lead to fresh-water contact in schistosomiasis-endemic areas and other risk behaviors	Women or girls who are living or have lived in or near schistosomiasis-endemic areas	To identify women or girls at risk of FGS	During routine household visits or at community health outreach events	Knowledge of FGS risk factors (i.e. fresh-water contact behavior)
**2.1c**	Community health worker/ peer educator	Asks questions in a gender and culturally sensitive manner about the presence of typical symptoms[2] of FGS such as vaginal discharge, contact bleeding, history of STIs that do not respond to treatment, and infertility	Women or girls who are living or have lived in or near schistosomiasis-endemic areas	To identify women or girls at risk of FGS	During routine household visits or at community health outreach events which specifically target women or perform outreach where women typically congregate in the community	Knowledge of symptoms and ability to address privacy and confidentiality concerns
**2.1d**	Community health worker/ peer educator	Asks questions in a gender and culturally sensitive manner about any previous history of urinary schistosomiasis or history of STIs that do not respond to treatment	Women or girls who are living or have lived in or near schistosomiasis-endemic areas	To identify women or girls at risk of FGS	During routine household visits or at community health outreach events which specifically target women or perform outreach where women typically	Knowledge of FGS risk factors, symptoms, and knowledge of incorrect diagnosis of STIs

					congregate in the community	
**2.1e**	Community health worker/ peer educator	Identifies communities or community members who do not have access or do not benefit from mass drug administration (if MDA is ongoing) such as school-age children who are not in school, adults at risk, and marginalized individuals (e.g. people living with disabilities, migrants, indigenous groups, etc.)	Community leaders or local opinion leaders and women or girls who are living or have lived in or near schistosomiasis-endemic areas	To identify women or girls at risk of FGS	During routine household visits, at community health outreach events or MDA records (where available)	Awareness of equity gaps in own community and knowledge of how to obtain praziquantel for schistosomiasis and FGS prevention
**2.1f**	Community health worker/ peer educator	Recognizes the symptoms of female genital schistosomiasis, provides counseling, and refers to a local clinic for treatment and potential pelvic examination or colposcopy after women describe symptoms of FGS/STIs	Women describing symptoms of urinary schistosomiasis or FGS/STIs	Referral to the community clinic in order to reliably treat women at the health facility	Interacting at the community level during routine household visits or at community health outreaches	Knowledge of FGS risk factors, symptoms, and knowledge of referral options for patients to get treatment and further diagnostic work up. Knowledge of FGS-associated stigma and the local socio-cultural context.
**2.2 Prevent schistosomiasis and FGS**						
**#**	**Who**	**Does what**	**To Whom**	**For what**	**When**	**Required Knowledge**

**2.2a**	Community health worker/ peer educator	Delivers health education about preventing schistosomiasis and FGS including avoidance of contact with fresh water sources in schistosomiasis -endemic areas and other risk factors, and taking part in mass drug administration (MDA) in the region	At-risk communities (according to guidelines) including women and girls	To prevent schistosomiasis and FGS	During routine household visits or at community health outreach events in schistosomiasis -endemic and surrounding communities	Trained to deliver health education information and knowledgeable about risk factors and prevention of schistosomiasis and FGS
**2.2b**	Community health worker	Correctly administers praziquantel (PZQ) using a dose pole as a single treatment during community MDA	At-risk communities (according to guidelines) including women and girls	To prevent and treat schistosomiasis	During community MDA activities	Trained to deliver MDA for schistosomiasis
**2.2c**	Community health worker/ peer educator	Delivers treatment or information on where to access praziquantel outside of MDA	Anyone, especially women or girls, who have not benefited from MDA with or without symptoms	To prevent and treat schistosomiasis	During routine household visits or at community health outreach events in schistosomiasis-endemic and surrounding communities	Knowledgeable about risk factors and prevention of schistosomiasis and FGS and referral options for accessing PZQ. Knowledgeable about the need for repeated treatment with PZQ or regular administration of MDA for individuals living in endemic areas with poor water & sanitation facilities.

**2.2d**	Community health worker /peer educator	Informs and counsels women and girls FGS poses an increased risk of HIV acquisition if exposed to HIV and any other chronic complications of FGS such as infertility and sub-fertility	Women and girls in schistosomiasis-endemic communities	To prevent schistosomiasis and HIV	During routine household visits or at community health outreach events in schistosomiasis-endemic and surrounding communities	Knowledgeable about schistosomiasis/FGS and link between FGS and HIV acquisition and awareness and sensitivity around potential stigma with that association
**2.2e**	Community health worker/ peer educator	Delivers health education and counseling on symptoms of FGS and overlap with symptoms of STIs and cervical cancer	Women in schistosomiasis-endemic communities and their partners	To prevent FGS and proper treatment for FGS, STI and cervical cancer	During routine household visits or at community health outreach events in schistosomiasis-endemic and surrounding communities	Knowledgeable about the symptoms of FGS, STIs, and cervical cancer
